# Deciphering germination rate differences in quinoa genotypes via integrated phenotypic, structural, and transcriptomic approaches

**DOI:** 10.1186/s12870-026-08676-7

**Published:** 2026-04-02

**Authors:** Jianxun Huang, Yulai Zhang, Xinhui Yang, Chengqi Yang, Yan Zhao, Jiayi Shi, Aixia Ren, Pengcheng Ding, Hafeez Noor, Xiangyun Wu, Sumera Anwar, Zhiqiang Gao, Min Sun, Linghong Li

**Affiliations:** 1 College of Agriculture, Key Laboratory of Sustainable Dryland Agriculture of Shanxi Province, Shanxi Agricultural University, Taiyuan, 030031 China; 2 Shanxi Provincial Key Laboratory of Crop Ecology and Efficient Water & Nutrient Use, Jinzhong, China; 3 College of Agronomy, Qingdao Agricultural University, Qingdao, 266109 China; 4 Shanxi Jiaqi Agri-Tech Co., Ltd., Taiyuan, 030006 China; 5 Department of Botany, Government College Women University, Faisalabad, Pakistan

**Keywords:** Germination mutants, Hormones, Seed coat structure, Transcriptome analysis, WGCNA

## Abstract

**Background:**

Quinoa (*Chenopodium quinoa* Willd.) is a nutritionally valuable and stress-tolerant crop in which seed germination plays a critical role in seedling establishment and yield formation. However, the integrated regulatory mechanisms underlying genotypic variation in germination are not yet fully understood. Most existing studies have focused on phenotypic traits or single-omics approaches, leaving a knowledge gap in systematic analyses that combine seed coat architecture, hormone dynamics, transcriptomics, and gene co-expression networks.

**Results:**

Phenotypic analysis showed that WT seeds achieved 100% germination at 12 h, while the moderately germination‑defective *TF* and the severely germination-defective *TS* mutants reached 92% and 29.33%, respectively. Seed coat characterization revealed that the palisade layer thickness was 13.31 μm in WT, increasing to 16.66 μm in *TF* and 20.07 μm in *TS*. Hormone dynamics monitoring indicated that GA₃ content in WT peaked at 1.859 ng·g⁻¹ at 8 h of imbibition, with a GA₃/ABA ratio of 0.53. In contrast, GA₃ levels in the *TS* mutant remained below 0.200 ng·g⁻¹ throughout, and the GA₃/ABA ratio stayed under 0.05. Transcriptomic and WGCNA analyses identified the Tan module, positively correlated with germination rate (*r* = 0.89), and the Black module, negatively correlated (*r* = − 0.60), and screened hub genes within the regulatory network. These results demonstrate that the coordinated actions of seed coat structure, hormone homeostasis, and transcriptional programming collectively determine the germination performance of different quinoa genotypes.

**Conclusions:**

This study provides multi-dimensional integrated data for deciphering the regulatory mechanisms of quinoa seed germination, and systematically clarifies how seed coat structure, hormone dynamics, and transcriptional networks synergistically regulate the germination process. These findings deepen our understanding of the biological basis of quinoa seed germination and establish a solid foundation for future functional studies and molecular improvement of germination-related traits in quinoa.

**Supplementary Information:**

The online version contains supplementary material available at https://doi.org/10.1186/s12870-026-08676-7.

## Introduction

Seed germination marks the initiation of the plant life cycle and is the first critical stage that determines crop establishment. Rapid and uniform germination promotes synchronized seedling emergence and optimal stand establishment, providing a solid foundation for achieving stable yields [1, 2]. Conversely, delayed or uneven germination often leads to poor seedling vigor and reduced competitiveness, particularly in challenging environments, where early establishment is crucial for survival [3, 4]. The variation in germination rate among phenotypes is not random, but functions as a key adaptive strategy shaped by environmental heterogeneity [5]. The fast-germinating phenotype can quickly occupy ecological niches in regions with short, favorable conditions, such as after transient rainfall in arid zones. In contrast, slow germination acts as a bet-hedging strategy, reducing the risk of seedling death under sudden drought, low temperature, or high salinity, by waiting for more stable conditions [6]. This ecological diversification underscores the importance of understanding the genetic and physiological determinants of germination rate, especially for crops such as quinoa, which are cultivated across a wide range of climatic conditions.

Quinoa (*Chenopodium quinoa* Willd.) is an annual pseudo-cereal domesticated in the Andean region 3,000–5,000 years ago [7]. It is renowned as a “complete food” due to its exceptionally high protein content, balanced essential amino acid profile, and rich micro-nutrient composition [8]. Beyond its nutritional superiority, quinoa possesses exceptional tolerance to multiple abiotic stresses, such as drought and salinity, making it a prime candidate for cultivation in less fertile and climate-vulnerable regions [9, 10]. Therefore, ensuring reliable cultivation is critical to global food security. As a typical halophyte and stress-tolerant crop, quinoa exhibits unique physiological and molecular characteristics during seed germination, and its germination efficiency is jointly regulated by genetic, physiological, and environmental factors [11–13]. Compared with conventional crops, quinoa activates a series of key pathways involved in osmotic regulation, reactive oxygen species scavenging, cell wall modification, and energy metabolism at the early stage of germination, which provides a basis for maintaining a high germination rate under stress conditions [14]. Multiple studies have shown that the gene expression during quinoa germination presents obvious stage-specificity and genotypic differences. For example, under salt stress, quinoa seeds can rapidly induce the expression of genes related to ion transport, synthesis of osmoprotective substances, and antioxidant enzyme systems, thereby alleviating stress damage [15]. Under low-temperature germination conditions, quinoa upregulates genes related to membrane lipid metabolism, sugar metabolism, and cold-responsive transcription factors to maintain cell membrane stability and ensure energy supply [16].

The morphological characteristics of seed dormancy and germination include the shape and size of the embryo, as well as the biophysical properties of the seed coat, endosperm, and other tissues that may confer dormancy characteristics to the seed coat [17–20]. The seed coat regulates water uptake and gas exchange, and imposes mechanical constraints, with its thickness and structure directly influencing dormancy intensity [6]. In quinoa, accessions with thicker or more prominent seed coats consistently exhibited higher dormancy [21].

Seed germination is influenced by internal hormones. For example, hormones such as gibberellin (GA), abscisic acid (ABA), ethylene (ET), auxin (AUX), cytokinin (CTK), brassinolide (BR), and jasmonic acid (JA) are key endogenous factors that regulate seed germination [22–29]. Hormonal signalling is the second major regulatory layer and is primarily mediated by the balance between ABA and GAs. ABA acts as the principal germination inhibitor, maintaining dormancy, whereas GAs promote germination by facilitating endosperm weakening and embryo expansion [30]. The dynamic shift in the ABA-to-GA ratio during imbibition is critical, dormancy release is regulated by ABA catabolism and GA biosynthesis [31]. The fast-germinating phenotype, as observed in certain tomato mutants, is often linked to reduced ABA content or sensitivity [32]. This process is not controlled by a single pathway, but involves a complex network in which transcription factors interact with hormonal signals to coordinately regulate seed development and dormancy [33, 34].

To address these gaps, we analyzed wild-type (WT) quinoa and two ethyl-methane sulfate-derived mutants *TF* and *TS*, the moderately germination-defective *TF* germinates faster than the severely germination-defective *TS* mutant, but both are slower than the wild type. Our central hypothesis is that differences in germination speed among these quinoa genotypes arise from the combined effects of seed coat structural properties and differential expression of hormone- and metabolism-related genes, particularly those involved in ABA/GA balance and starch degradation. The objectives of this study were to (i) compare the germination phenotypes of WT, *TF*, and *TS* under different conditions; (ii) Characterization of seed coat ultrastructural differences among the three lines; (iii) Construct a gene co-expression network related to germination efficiency and screen key regulatory hubs in quinoa.

## Materials and methods

### Experimental materials

Plant materials were provided by Shanxi Jiaqi Seed Industry Co., Ltd. Taiyuan, China. The wild-type quinoa variety JQ505 shows normal germination characteristics. Two mutant lines, designated *TF* (*moderate germination-defective mutant*) and *TS* (*severe germination-defective mutant*), were generated by ethyl methane sulfonate (EMS) mutagenesis as described previously [35]. *TF* germinates faster than *TS*, but both mutants germinate more slowly than the wild-type JQ505. Mutant lines have been propagated for at least three generations under controlled conditions and exhibited stable phenotypes. The genetic background was confirmed to be identical to that of the wild type using simple sequence repeat marker analysis [36].

Before the experiment, seeds were surface-sterilized by immersion in 75% ethanol for 30s, followed by three rinses with sterile water. Subsequently, the seeds were soaked in 0.1% (w/v) mercuric chloride for 5 min and rinsed thoroughly with sterile distilled water five times to remove residual disinfectant. The disinfected seeds were placed in Petri dishes lined with two layers of filter paper and moistened with 5 mL of sterile distilled water. Three biological replicates were prepared for each genotype (WT, TF, and TS), with 50 seeds per replicate. Seeds were incubated in a growth chamber at 25 °C under a 16h light/ 8h dark photoperiod and 90% relative humidity. These conditions were selected according to optimal germination parameters reported for quinoa [37].

### Seed germination and water absorption rate determination

Germination was monitored at 0, 4, 8, 12, 24, and 48 h after sowing, seeds were considered germinated when the radicle protruded by more than 2 mm, and germination rate was calculated as the percentage of germinated seeds relative to the total number of seeds tested. For water absorption rate determination, seeds were weighed accurately before sowing and at each monitoring time point. Surface water was blotted with filter paper prior to weighing. Water absorption rate was calculated as [(Fresh weight - Initial weight)/Initial weight] × 100. Three germination vigor indices were calculated, including time to 50% germination (T₅₀) via linear interpolation, mean germination time (MGT) as Σ(Gₜ×Tₜ)/ΣGₜ, and germination index (GI) as Σ(Gₜ/Tₜ), where Gₜ is the number of germinated seeds at time Tₜ, and Tₜ is the corresponding incubation time (h).

### Seed coat ultrastructure observation

For transmission electron microscopy (TEM), seeds of WT, *TF*, and *TS* were cut transversely at approximately one-third of the distance from the embryo end. The cut seeds were immediately fixed in 2.5% glutaraldehyde solution (pH 7.2) at 4 °C for 24 h, rinsed in 0.1 M phosphate-buffered saline (PBS pH 7.2), and then post-fixed in 1% osmium tetroxide (OsO_4_) for 2 h at room temperature. The samples were dehydrated using ethanol gradient solutions (30%-100%) for 15 min each. Then, After sequential infiltration (ethanol: resin = 3:1, 1:1, 1:3, each for 2 h), seeds were embedded in Spurr’s resin and polymerized at 70 °C for 8 h to form solid blocks for sectioning.

Ultrathin sections (~ 70–90 nm) were obtained using a Leica EM UC7 ultramicrotome (Leica Microsystems, Germany). The sections were placed on copper grids and stained with uranyl acetate and lead citrate. The sections were observed using a Hitachi H-7650 transmission electron microscope (Hitachi High-Technologies, Japan) operating at 80 kV. Five fields of view per genotype were imaged to ensure representation. TEM images were analyzed using Image-Pro Plus 6.0 software (Media Cybernetics, USA). Structural parameters, including the thickness of the seed coat, epidermis, palisade layer, and parenchymal cell layer were measured [38]. At least ten independent measurements were performed for each parameter.

### Measurement of ABA and GA_3_ content

The samples preserved at a temperature of -80 °C were promptly pulverized into powder using liquid nitrogen, and the ABA and GA_3_ contents were quantified by the HPLC-ESI-MS/MS internal standard method [39]. Standard curves were established for quantitative calculation. The GA_3_/ABA ratio was calculated to evaluate hormone balance. Each genotype had three biological replicates.

### Exogenous hormone and abiotic stress treatments

Seeds of each genotype were subjected to four exogenous treatments with distilled water as control. Optimal concentrations determined by preliminary experiments were used including 100 µM ABA, 200 µM GA₃, 150mM NaCl, 10% PEG-6000. Experimental design was consistent with the germination test with three biological replicates per treatment. Seeds were cultured in Petri dishes saturated with corresponding treatment solutions. Germination status was recorded at 0, 4, 8, 12, 24, and 48 h. Germination rates were calculated and statistically compared among genotypes and treatments.

### Transcriptome sequencing and analysis

For transcriptomic analysis, WT, *TF*, and *TS* seeds were sampled at 0 h, 12 h, and 24 h post-germination, with three biological replicates per time point. Samples were immediately frozen in liquid nitrogen and stored at -80 °C. Total RNA was isolated using the Trizol Reagent (Invitrogen). Its concentration, quality and integrity were assessed via the NanoDrop spectrophotometer (Thermo Scientific). RNA samples were used for library construction. mRNA was purified with poly-T oligo-attached magnetic beads, fragmented, and reverse-transcribed into cDNA. Libraries were prepared by adaptor ligation, size selection (400–500 bp, AMPure XP system), and PCR enrichment, then sequenced on the NovaSeq 6000 platform (Illumina) by Shanghai Personal Biotechnology Co., Ltd.

Raw reads were filtered with Cutadapt to remove adapters and low-quality sequences (≥ 20% adapter overlap or mean quality < Q20). Clean reads were aligned to the quinoa reference genome (*Chenopodium quinoa* Willd., NCBI build GCF_001683475.1_ASM168347v1) using HISAT2 v2.2.1 [40]. Read counts were obtained using FeatureCounts v2.0.1 [41] the expression values were normalized to FPKM. Differentially expressed genes (DEGs) were identified using DESeq2 v1.38.3[42]. The filtering criteria for DEG calling were set as |log2FC| ≥ 1 and adjusted *P*-value (FDR) ≤ 0.05, which are widely accepted and robust thresholds in RNA-seq differential expression analysis to balance the identification of genuine biological differences and reduction of false positives. Dispersion of gene expression levels was estimated using the default statistical model in DESeq2, a standard approach for minimizing technical noise and optimizing the reliability of differential expression calls. Sequencing quality was monitored by evaluating base quality distribution, GC content stability, and read error-rate profiles throughout the analysis.

### WGCNA analysis

Prior to WGCNA, sample quality was assessed using hierarchical clustering. Most samples clustered according to genotype and time point as expected, but two samples (A12hTF_Y1 and 12hW_Y2) deviated significantly from their respective biological replicate groups and showed low correlation with others. These two outliers were excluded to ensure network robustness, leaving 19 samples for downstream analysis.

Weighted gene co-expression network analysis (WGCNA) was performed using the WGCNA package in R 4.5.2 [43]. Low-expression genes and non-differentially expressed genes were filtered out to reduce technical noise. The top 80% of genes with the highest expression variation were retained using the Genefilter package, enriching for biologically responsive genes and improving network stability. A total of 8000 genes were used for network construction. The optimal soft-threshold power (β = 10) was selected using the pickSoft Threshold function to approximate a scale-free topology. Genes with similar expression patterns were clustered into nine modules. Correlations between module eigengenes and physiological traits were calculated to identify germination-associated modules. Gene Ontology [44] and KEGG [45] enrichment analyses were performed to annotate the biological functions of each module. Hub genes were screened based on module eigengene‑based connectivity (kME), and networks were visualized using Cytoscape 3.10.4 [46],with highly connected and highly expressed genes defined as core hub genes. To evaluate the robustness of module–trait associations, bootstrap resampling (1000 iterations) was performed, and 95% BCa confidence intervals were calculated using the R package boot (v1.3.32). 

### Validation of gene expression by qRT-PCR

Eight DEGs related to germination were selected for qRT-PCR validation. Primers were designed using Primer Premier 5.0 (Premier Biosoft, USA), targeting 100–200 bp amplicons with a melting temperature of ~ 60 °C (listed in Table S1). *Actin *was used as an internal reference gene. The reaction mixture (20 µL) contained 10 µL SYBR Green Master Mix (TaKaRa, Japan), 2 µL cDNA, 0.5 µL of each primer (10 µM), and 7 µL RNase-free water. Amplifications were performed on a Bio-Rad CFX96 real-time PCR system under the following conditions: 95 °C for 30 s, followed by 40 cycles of 95 °C for 5 s and 60 °C for 30 s. Melting curve analysis confirmed amplification specificity. Relative expression was calculated using the 2^(− ΔΔCt) method [47]. Each reaction was performed in triplicates.

### Partial Least Squares Path Modeling (PLS-PM) analysis

To further explore causal and predictive relationships among seed coat traits, hormone ratio (GA₃/ABA), module eigengenes, and germination rate, partial least squares path modeling (PLS‑PM) was performed using the plspm package in R. The model included seed coat traits and hormone ratio as exogenous variables, four key co‑expression modules (MEpink, MEtan, MEblack, MEgrey) as mediators, and germination rate as the final endogenous variable. All variables were standardized prior to analysis. Path coefficients and 95% CIs were estimated using bootstrap resampling with 1000 iterations. A path was considered significant if its 95% CI did not include zero. Model performance was evaluated using the coefficient of determination (R²) for each endogenous variable.

### Statistical analysis

Statistical analyses were performed using R version 4.5.2, with significance set at *P* < 0.05. All data are presented as means ± standard deviation (SD). For seed coat anatomical parameters, one-way analysis of variance (ANOVA) followed by Tukey’s HSD test was used to compare differences among genotypes, with distinct letters indicating significant differences (*P* < 0.05). Pearson correlation analysis was conducted to assess the relationship between water absorption rate and GA₃/ABA contents within each genotype. For transcriptomic data, a heatmap of the top 300 most variable DEGs was generated using the pheatmap package, based on row-wise Z-score normalization of averaged biological replicates and hierarchical clustering.

## Results

### Seed germination characteristics and water absorption rate changes

**Fig. 1:**
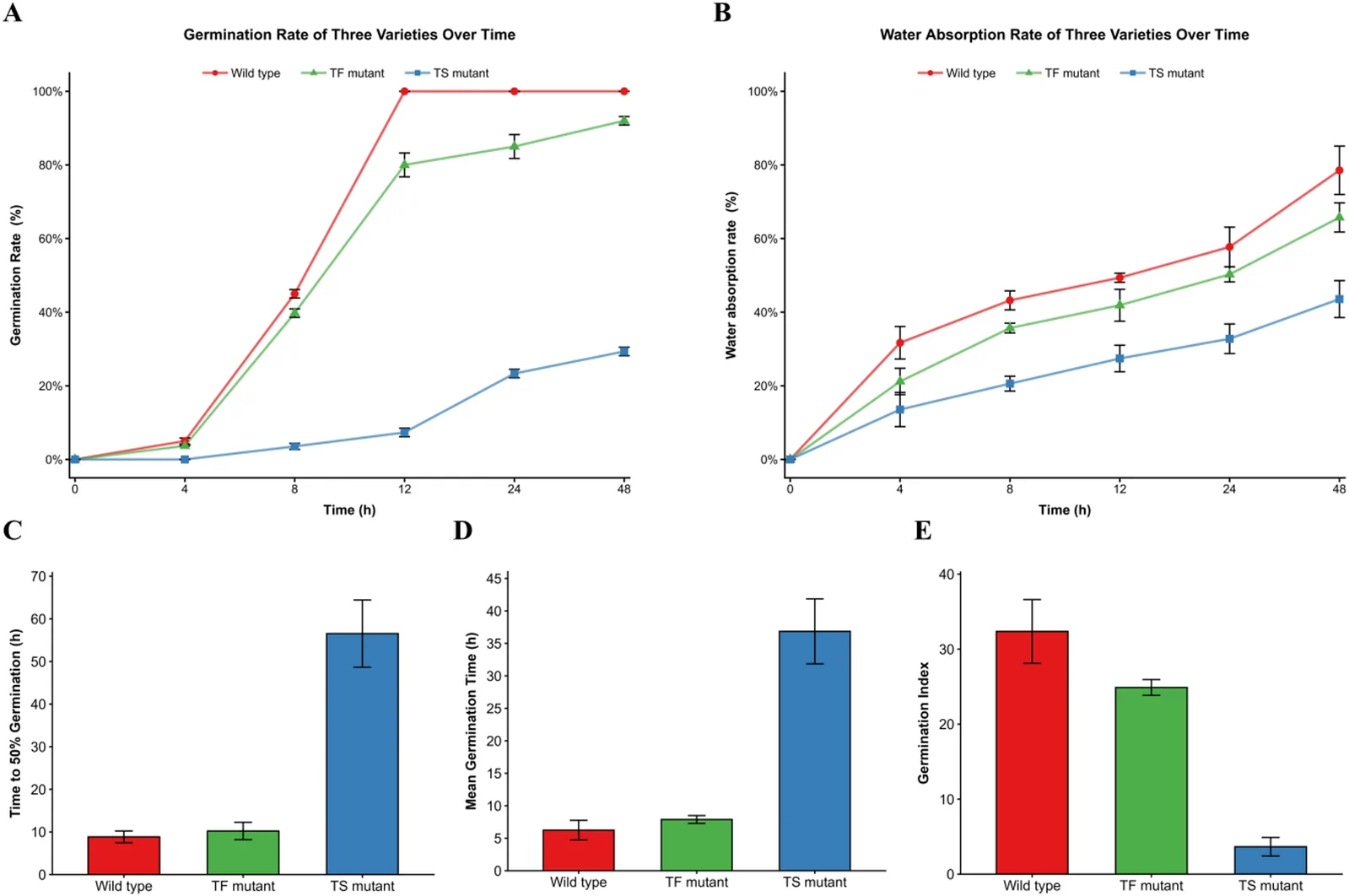
Seed germination characteristics and water absorption rate changes. **A** Germination rate and (**B**) Water absorption rate of WT, *TF*, and *TS* mutants over times. **C** Time to 50% germination (T₅₀), (**D**) Mean germination time (MGT), and (**E**) Germination index (GI) of WT, *TF*, and *TS* mutants

To evaluate the germination characteristics and water absorption properties of the wild type, *TF* mutant, and *TS* mutants, dynamic changes of germination rate and water absorption rate were monitored over a 48-hour period, along with germination vigor indices T₅₀, MGT, and GI. The wild type exhibited the earliest germination initiation and fastest germination progression, with a germination rate of 5.00% at 4 h and reaching 100% by 12 h, which was maintained until 48 h. In contrast, the *TF* mutant showed a delayed germination process, with a germination rate of only 3.69% at 4 h, 80.00% at 12 h, and 92.00% at 48 h. The *TS* mutant displayed the most severely retarded germination, with no germination observed within 4 h. Its germination rate was merely 3.54% at 8 h, 7.33% at 12 h, and only 29.33% even at 48 h (Fig. 1A).

The water absorption rates increased over time of all three genotypes, but significant genotypic differences were observed. The wild type exhibited the highest water absorption rate, reaching 31.69% at 4 h and 78.54% at 48 h. In comparison, the *TF* mutant showed a lower water absorption capacity, with values of 21.18% at 4 h and 65.73% at 48 h. Among the three genotypes, the *TS* mutant displayed the lowest water absorption rate, recording only 13.55% at 4 h and 43.56% at 48 h (Fig. 1B). Consistent with the germination and water absorption patterns, germination vigor indices also varied distinctly across genotypes. The wild type showed the shortest T₅₀ (8.85 h) and MGT (6.25 h), along with the highest GI (32.36). In the *TF* mutant, T₅₀ was extended to 10.23 h, MGT increased to 7.89 h, and GI decreased to 24.89. Notably, the *TS* mutant exhibited the longest T₅₀ (56.54 h) and MGT (36.85 h), accompanied by the lowest GI (3.65) (Fig. 1C, D, E).

### Seed coat ultrastructure of different genotypic quinoa seeds

**Fig. 2:**
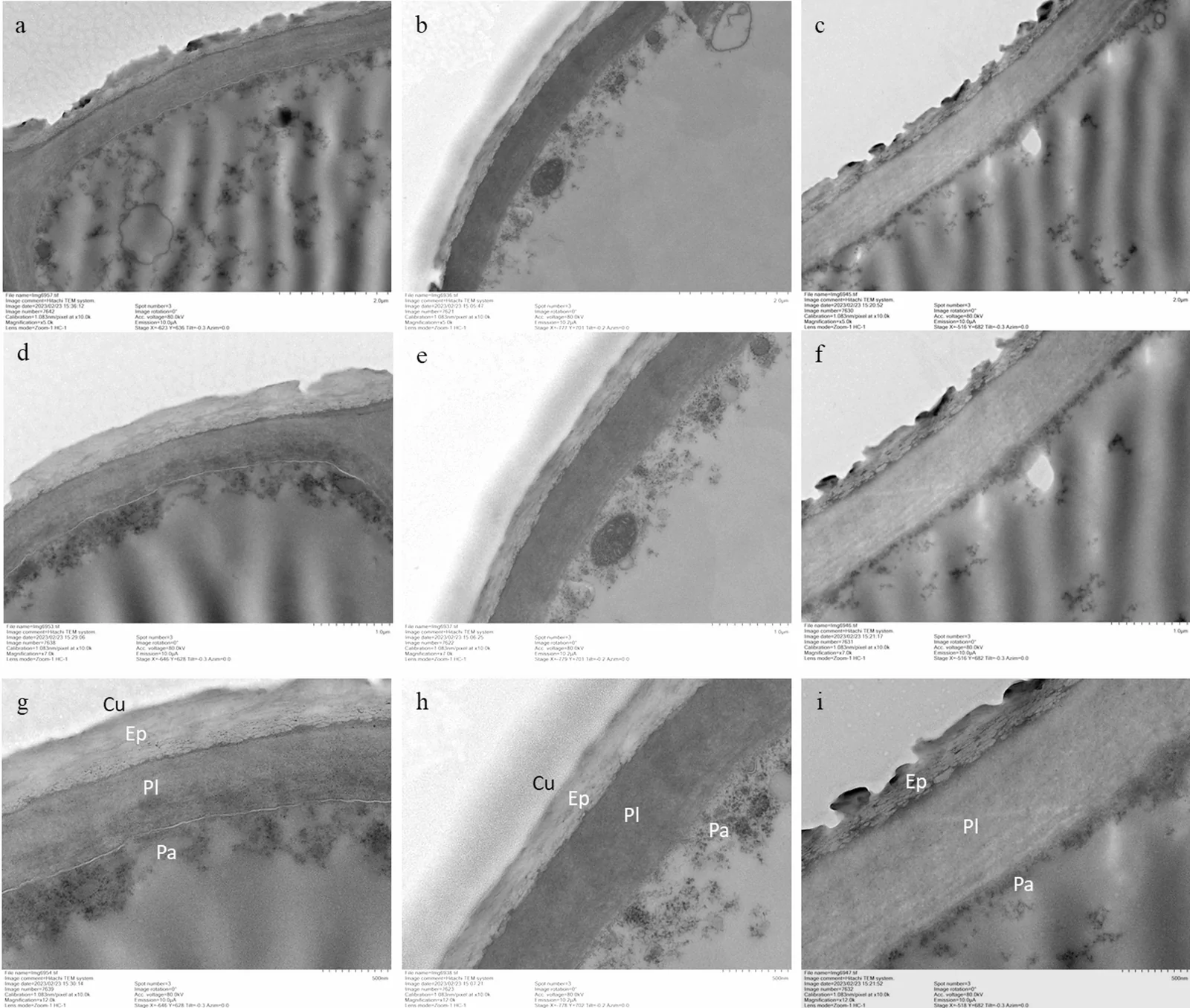
Transmission electron microscopy (TEM) images of quinoa seed coats from wild type and germination mutants. Panels **A**, **D**, **G** show the wild type; **B**, **E**, **H** show the *TF* mutant; and **C**, **F**, **I** show the *TS* mutant. Scale bars represent: 2 μm in **A**–**C**, 1 μm in **D**–**F**, and 500 nm in **G**–**I**. Cu: cuticle; Ep: epidermis; Pl: palisade; Pa: parenchyma

Transmission electron microscopy (TEM) observations revealed that the quinoa seed coat consists of a thin cuticle, epidermis, palisade layer, and inner parenchymatous layer (Fig. 2). The wild-type (WT) seed coat displayed well-defined layers, with a relatively thin palisade layer and an intact cuticle (Fig. 2A, D, G). In the *TF* mutant, the epidermis was thinner, the palisade layer appeared irregular in structure, and the parenchymatous layer was thicker (Fig. 2B, E, H). In contrast, the *TS* mutant exhibited large, irregular intercellular spaces within the palisade layer and a thinner parenchymatous layer (Fig. 2C, F, I).

Quantitative measurements of seed layer thickness revealed significant differences among the WT, *TF* mutant, and *TS* mutant (Table 1). The overall seed coat was thickest in the *TF* mutan (36.20 μm), significantly exceeding that of the WT (32.35 μm) and the *TS* mutant (31.34 μm). The epidermis was thickest in the WT (9.25 μm), significantly thicker than in the *TF* (6.78 μm) and *TS* (6.75 μm) mutants. In contrast, the palisade layer was thickest in the *TS* mutant (20.07 μm), followed by the *TF* mutant (16.66 μm) and the WT (13.31 μm). Notably, the palisade layer in the *TF* mutant was significantly thicker than that in the WT. Conversely, the parenchyma/endodermis cell layer was thickest in the *TF* mutant (11.78 μm), significantly thicker than that in the WT (9.82 μm), and thinnest in the *TS* mutant (4.94 μm). Parenchyma cells were loosely arranged with visible intercellular spaces in both the WT and *TF* mutant, whereas in the *TS* mutant, parenchyma cells adjacent to the palisade layer were densely packed.

**Table 1:** Thickness of seed coat layers in wild type and germination mutants of quinoa as observed under transmission electron microscopy (µm)

Genotypes	Seed coat	Epidermal layer	Palisade layer	Parenchyma cell layer
Wild type	32.35 ± 0.75 b	9.25 ± 0.57 a	13.31 ± 0.60 c	9.82 ± 0.54 b
*TF* mutant	36.20 ± 1.46 a	6.78 ± 0.80 b	16.66 ± 0.49 b	11.78 ± 0.55 a
*TS* mutant	31.34 ± 1.11 b	6.75 ± 0.33 b	20.07 ± 0.53 a	4.94 ± 1.26 c

Values represent mean ± standard error (*n* = 10). Different lowercase letters (a, b, c) within a column indicate statistically significant differences (*P* < 0.05) according to Tukey’s HSD

### Endogenous hormone content changes

**Fig. 3:**
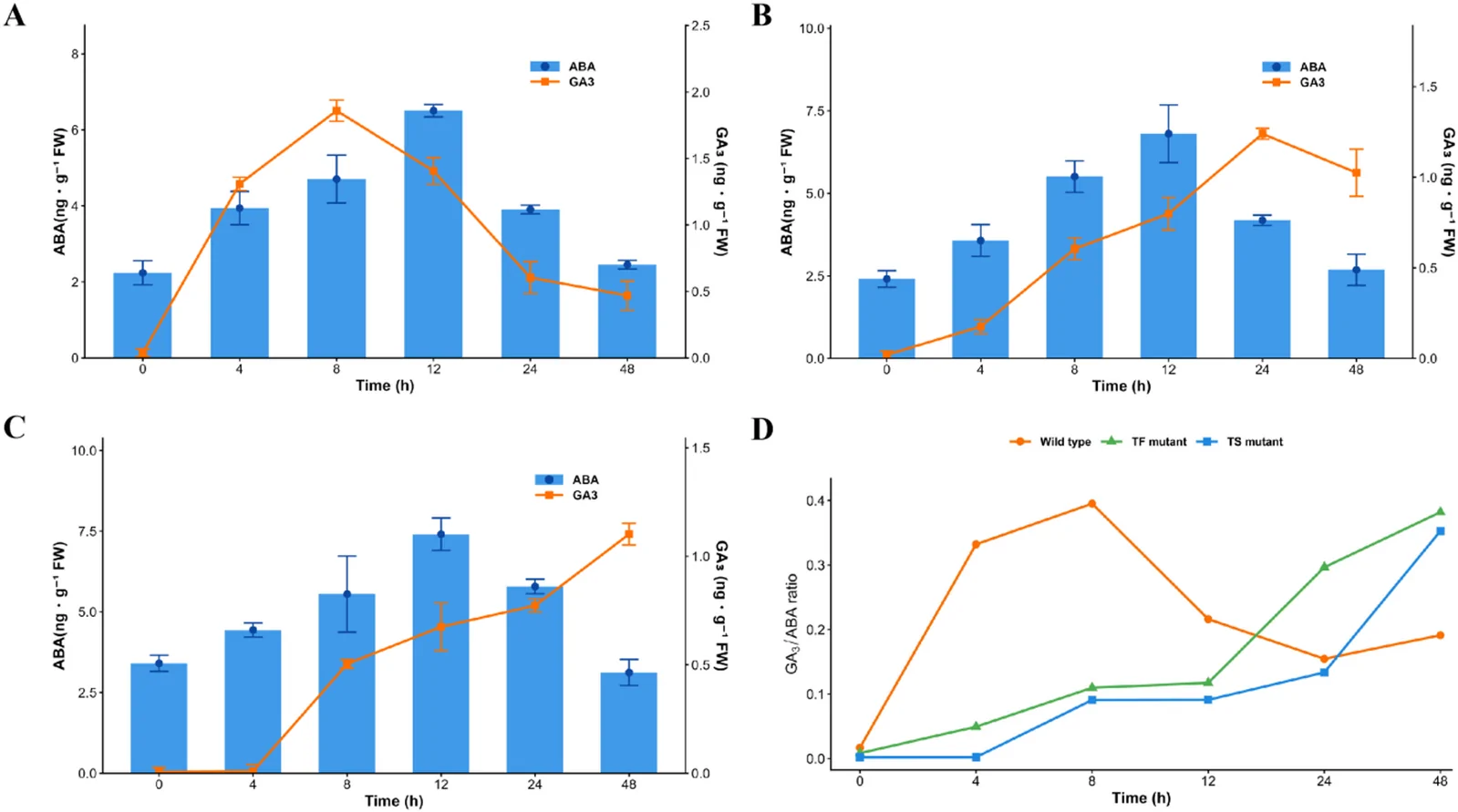
Changes in endogenous hormone ABA and GA_3_ content during the germination process of (**A**) wild type, (**B**) *TF* mutant, and (**C**) *TS* mutant quinoa seeds. **D** GA_3_/ABA ratio

Endogenous GA₃ and ABA contents in the three genotypes were quantified during 0–48 h of imbibition (Fig. 3). In wild-type seeds, GA₃ increased rapidly from 0.038 ng·g⁻¹  at 0 h to a peak of 1.859 ng·g⁻¹ at 8 h, then gradually declined and stabilized after 12 h; meanwhile, ABA content rose from 2.240 ng·g⁻¹ to a peak of 6.504 ng·g⁻¹  at 12 h before decreasing to 2.453 ng·g⁻¹  by 48 h (Fig. 3A). In the TF mutant, GA₃ accumulation was delayed, reaching only 0.799 ng·g⁻¹  at 12 h and peaking at 1.240 ng·g⁻¹  at 24 h, which represented 66.7% of the wild-type peak. Concurrently, ABA levels were slightly higher than in the wild type, starting at 2.408 ng·g⁻¹ , peaking at 6.806 ng·g⁻¹  (12 h), and declining slowly to 2.684 ng·g⁻¹  at 48 h (Fig. 3B). In contrast, the TS mutant exhibited consistently low GA₃ levels throughout imbibition, increasing only marginally from 0.007 ng·g⁻¹  to 1.102 ng·g⁻¹ by 48 h without a distinct peak. Meanwhile, ABA content remained the highest among the three genotypes, rising from 3.408 ng·g⁻¹  to a peak of 7.407 ng·g⁻¹  at 12 h, then decreasing gradually to 3.124 ng·g⁻¹  by 48 h (Fig. 3C). Similarly, the GA₃/ABA ratio peaked at 8 h in the WT, reached a delayed and lower peak at 24 h in the TF mutant, and remained consistently low throughout the imbibition period in the TS mutant (Fig. 3D). These hormone dynamics are consistent with the observed germination phenotypes across the three genotypes.

### Seed germination responses to exogenous hormones and abiotic stress

To further explore the regulatory role of hormones in seed germination and their interaction with environmental stress, seeds were treated with 100 μM ABA, 200 μM GA₃, 150 mM NaCl, and 10% PEG-6000, with distilled water as control (CK) (Fig. S1). Exogenous ABA suppressed germination, with inhibition intensity negatively correlated with endogenous ABA levels. The WT, having the lowest endogenous ABA, was most sensitive, exhibiting a 37.60% decrease in final germination rate (62.40% vs. 100.00%). The TF mutant showed a moderate reduction of 33.30% (58.70% vs. 92.00%). In contrast, the TS mutant, with the highest endogenous ABA, was least affected, showing only a 6.73% decline (22.60% vs. 29.33%) (Fig. S1A). Exogenous GA₃ effectively promoted germination. While the WT displayed accelerated early germination while maintaining 100% final germination (same as CK), the TF mutant increased by 6.50% (98.50% vs. 92.00%). Notably, the TS mutant showed a 16.47% improvement (45.80% vs. 29.33%), substantially alleviating its germination defect (Fig. S1B).

NaCl‑induced osmotic stress inhibited germination in all genotypes, with genotype‑specific tolerance observed. The WT exhibited the strongest tolerance, showing only a 10.50% reduction in final germination rate (89.50% vs. 100.00% in CK). The TF mutant displayed moderate sensitivity, with a 13.80% decrease (78.20% vs. 92.00%), while the TS mutant was the most sensitive, showing a 15.63% decline to 29.33% (Fig. S1C). 10% PEG‑6000-simulated drought stress caused similar but stronger inhibition in the mutants. The WT maintained a high final germination rate of 85.30% (14.70% lower than CK). The TF mutant showed a marked 19.40% reduction (72.60% vs. 92.00%), and the TS mutant dropped to 15.40% (Fig. S1D). 

### Transcriptomic analyses

**Fig. 4:**
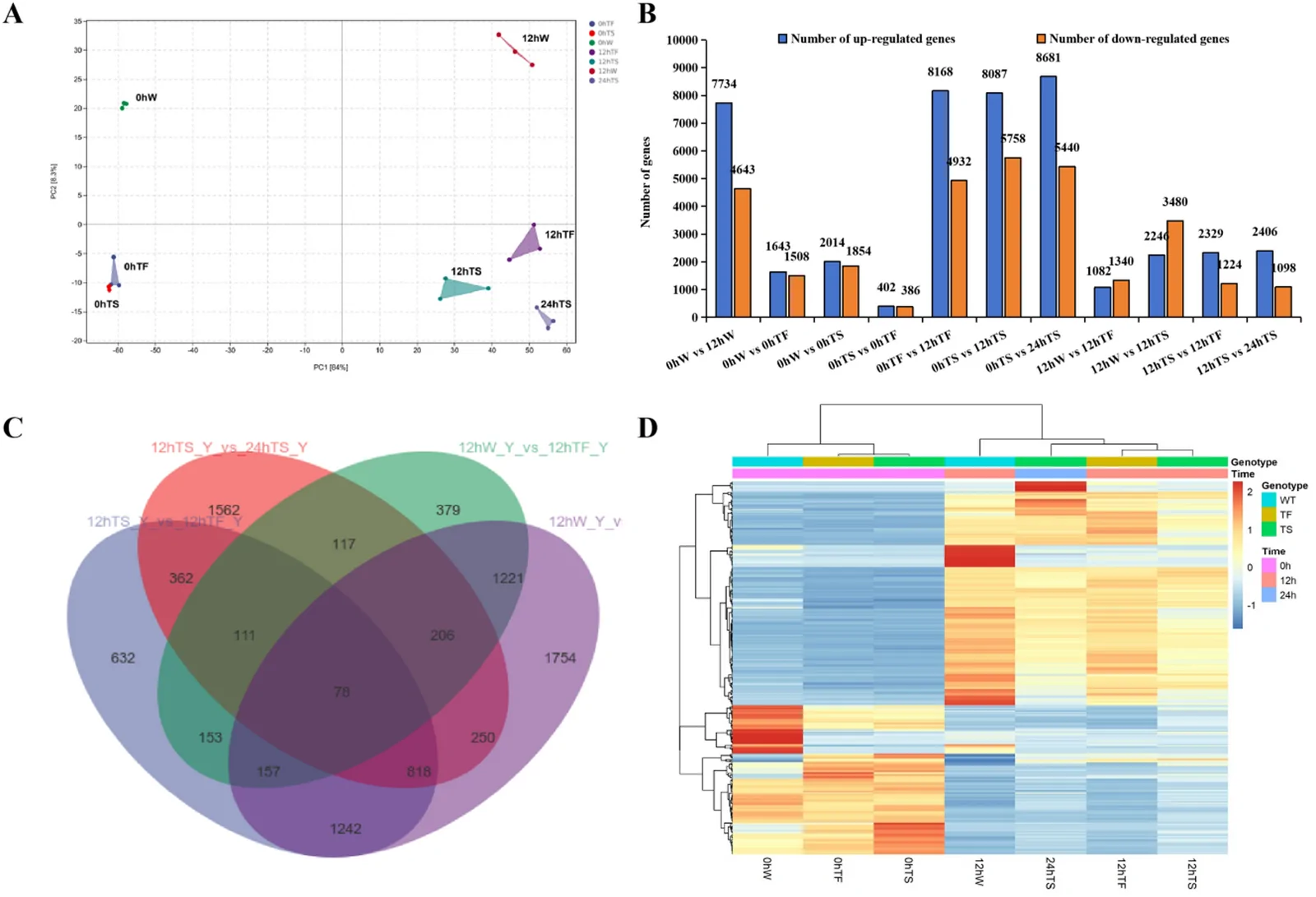
Correlation and PCA analysis of all transcripts and identification of DEGs. **A** PCA of transcriptome profiles. **B** Number of DEGs i*n each* comparison. **C** Venn diagram of DEGs among 4 comparison groups (FDR < 0.01 and FC ≥ 2). **D** Hierarchical clustering of the top 300 variable DEGs. Expression values were normalized by row Z-score, red and blue indicate up- and downregulation, respectively

RNA sequencing yielded over 33 million clean reads per sample, with Q30 scores exceeding 91.97% and uniquely mapped reads ranging from 94.41% to 96.21% (Table S2). These results indicate that the sequencing data were of high quality and suitable for transcriptomic analyses. Principal component analysis (PCA) was performed to evaluate expression patterns across genotypes and time (Fig. 4A). PC1 and PC2 accounted for 84.0% and 8.3% of the total variance, respectively. A clear separation was observed from 0 h to later stages along PC1. Genotypic differences were observed at each time point. For example, at 0 h, both *TF* and *TS* mutants clustered closely together and were distinct from WT. By 12 h, WT samples diverged markedly from both mutants, suggesting gradual, genotype-specific temporal shifts in gene expression.

Differential gene expression analysis revealed dynamic transcriptional changes across genotypes and germination stages (Fig. 4B). At 0 and 12 h, WT, *TF*, and *TS* exhibited 12,377, 13,100, and 13,845 DEGs, respectively. The *TS* mutant displayed 14,121 DEGs between 0 h and 24 h and 3,504 DEGs between 12 h and 24 h, suggesting that major transcriptional shifts occurred later in *TS* than in WT and *TF*. Comparative analysis of genotypes at individual time points revealed differences in gene expression patterns. At 0 h, 3,151 DEGs were observed between 0hW and 0hTF, 3,868 between 0hW and 0hTS, and 788 between 0hTS and 0hTF. At 12 h, the largest number of DEGs was detected between 12hW and 12hTS (5,726), followed by 12hTS vs. 12hTF (3,553) and 12hW vs. 12hTF (2,422). These results indicate that the distribution of DEGs demonstrated that gene expression changes were both time-dependent and genotype-specific. The complete expression profiles with functional annotations and DEG lists for all pairwise comparisons are presented in Table S3 and S4, respectively. Venn diagram analysis showed that only 78 genes overlapped in all four comparisons at 12 h (12hW vs. 12hTF, 12hW vs. 12hTS, 12hTS vs. 12TF), and 12 h vs. 24 h of *TS* (Fig. 4C). Similarly, only 92 DEGs were shared across genotypic comparisons (0hW vs. 0hTF, 0hW vs. 0hTS, and 0hTS vs. 0hTF) at 0 h (Fig. S2A). In contrast, 7,238 DEGs were conserved across time-course comparisons (0 h vs.. 12 h and 0 h vs. 24 h) in all genotypes (Fig. S2B). To visualize genome-wide expression dynamics, a heatmap of the top 300 most variable DEGs was generated (Fig. 4D). Hierarchical cluster analysis shows that quinoa of different times and different genotypes have different regulatory genes in the germination response, highlighting the gene-specific and stage-specific nature of gene expression during germination.

**Fig. 5:**
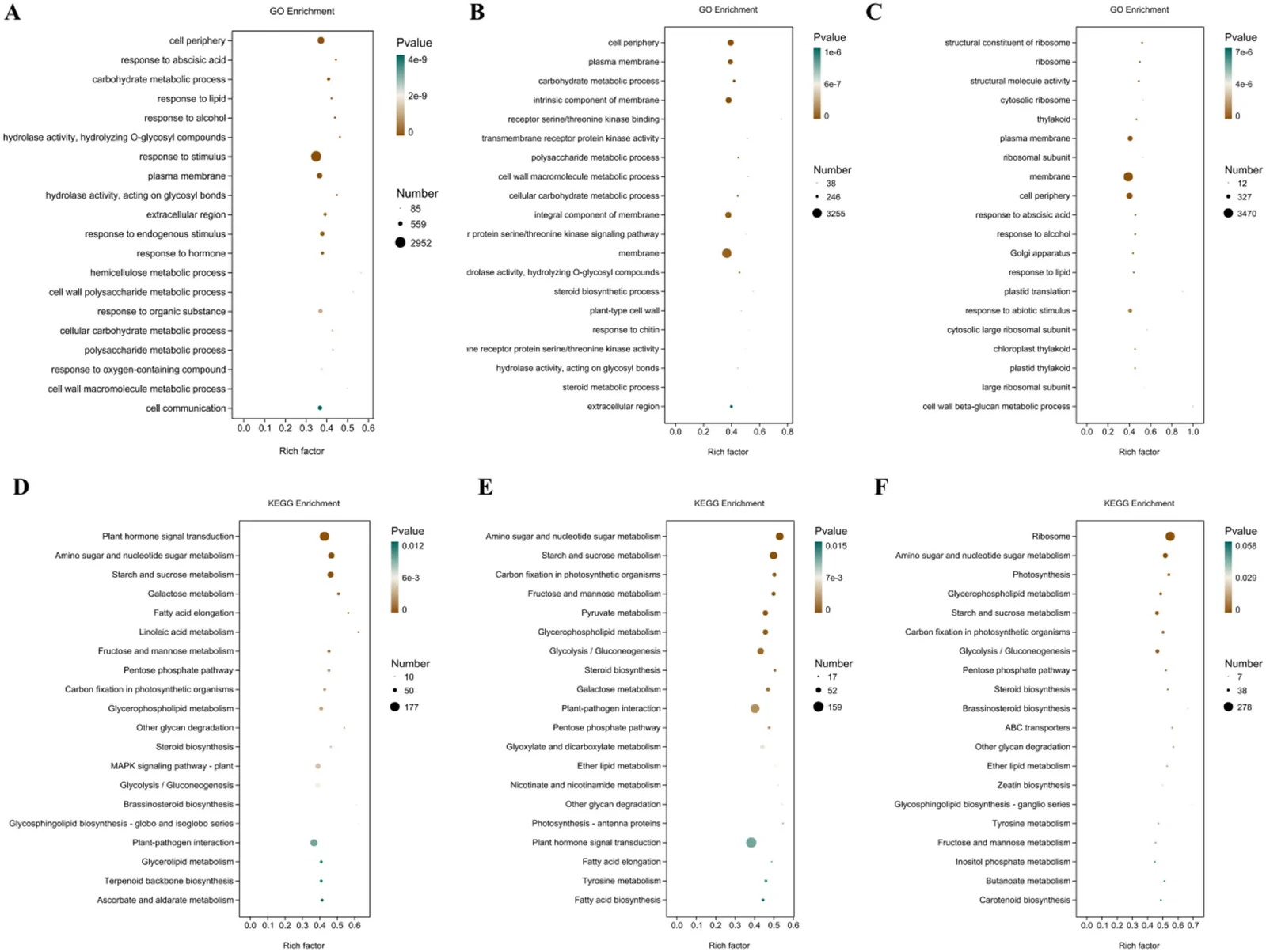
GO enrichment and KEGG pathway enrichment analysis of DEGs during seed germination. **A** GO and (**D**) KEGG enrichment of DEGs between 0 h and 12 h in the WT of quinoa. **B** GO and (**E**) KEGG enrichment of DEGs between 0 h and 12 h in the *TF* mutant. **C** GO and (**F**) KEGG enrichment of DEG between 0 h and 12 h in the *TS* mutant

To elucidate the biological mechanisms underlying the observed germination differences, we performed functional enrichment analysis and focused on pathways directly related to seed reserve mobilization, cell wall remodeling, and hormone signaling. In WT, DEGs were primarily enriched in hormone signal transduction (e.g., abscisic acid response) and carbohydrate metabolism (Fig. 5A), consistent with the active reactivation of metabolic pathways required for germination initiation. KEGG analysis confirmed that WT was characterized by key pathways such as starch and sucrose metabolism and plant hormone signaling (Fig. 5D), reflecting efficient reserve breakdown and hormonal balance. The *TF* mutant, which exhibits intermediate germination, showed a pronounced enrichment in cell wall modification (including “plant-type cell wall” in GO terms) and carbohydrate metabolism (Fig. 5B). KEGG analysis further highlighted enhanced amino sugar and nucleotide sugar metabolism (Fig. 5E), suggesting that *TF* is engaged in active cell wall loosening and polysaccharide remodeling—processes critical for radicle protrusion—although this occurs at a slower rate than in WT. In stark contrast, the severely delayed *TS* mutant displayed a distinctive enrichment pattern dominated by ribosomal biogenesis and plastid translation (Fig. 5C), with the ribosome pathway being the most significantly enriched KEGG term (Fig. 5F). This translational focus, coupled with the persistence of ABA response pathways and limited enrichment in starch metabolism, indicates that *TS* may be trapped in a stress-like state, prioritizing protein synthesis over the metabolic reprogramming necessary for germination. Additionally, pathways related to zeatin and carotenoid biosynthesis were enriched in *TS*, implying a dysregulation in phytohormone homeostasis that further impedes germination. GO and KEGG enrichment analyses for the direct comparisons (12hW_vs_12hTF, 12hW_vs_12hTS, and 12hTS_vs_12hTF) are presented in Fig. S3. Complete enrichment results for all comparisons are provided in Tables S5 and S6, respectively.

### Weighted correlation network analysis

**Fig. 6:**
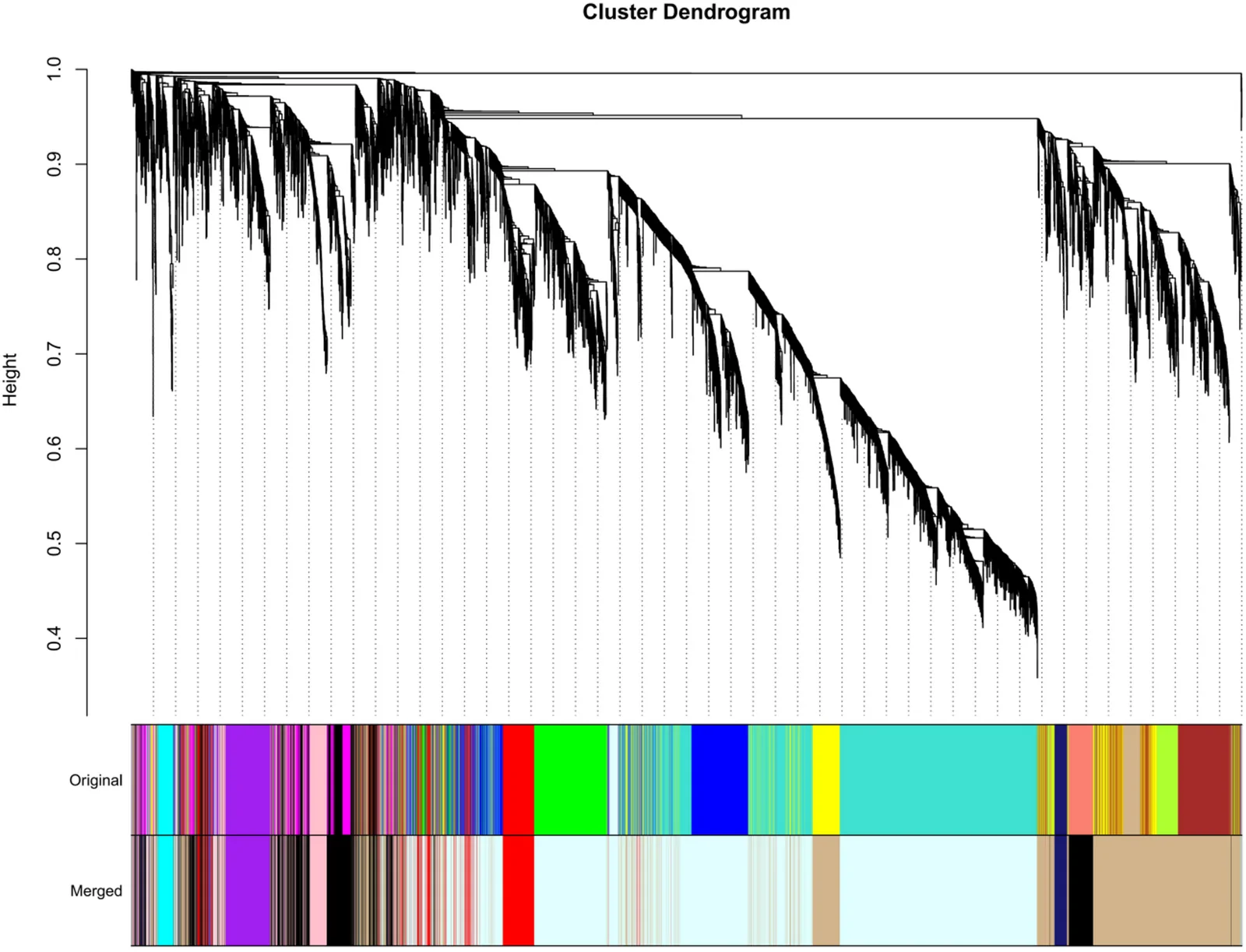
WGCNA tree clustering diagram of quinoa seed germination mRNA modules. The top hierarchical cluster dendrogram shows highly connected genes grouped into co-expression modules, with leaves representing individual genes and the height axis indicating clustering distance. The bottom panels Original and Merged display initial and merged module color assignments, respectively

After filtering to retain biologically informative and highly variable genes, a total of 8000 genes were used for WGCNA. Sample clustering was performed to remove outliers that could distort network topology, and two outlier samples were excluded. The remaining 19 samples formed consistent clusters (Fig. S4). The selection of appropriate soft‑threshold power is a critical step in constructing a weighted gene co‑expression network. A soft‑threshold power of 10 was chosen based on the criterion that it achieved a scale‑free topology fit index (*R*²) > 0.8, while the mean connectivity plateaued at approximately 305 (Fig. S5). These results confirm that the resulting network exhibits scale‑free distribution properties. Using the selected soft-threshold power (β = 10), a gene co-expression network was constructed and a clustering dendrogram was generated according to the correlation of gene expression patterns (Fig. 6). Ultimately, nine distinct gene modules were identified, each represented by a unique colour. The lightcyan module contained the highest number of genes (3,822), whereas the midnightblue module comprised the fewest (113 genes) (Tables S7).

Given the relatively small sample size (*n* = 19), bootstrap analysis of module membership yielded low Jaccard indices (mean < 0.1), indicating inherent variability in co-expression module boundaries under small-sample conditions. However, focused robustness analysis of module–trait associations yielded strong support for key modules (Fig. S6A). Bootstrap resampling (1,000 iterations) revealed that the 95% confidence intervals for correlations between eigengenes and germination rate did not include zero for MEpink, MEtan, MEblack, and MEgrey. These results demonstrate that the associations of these four modules with germination rate are statistically robust and not driven by sampling variation.

**Fig. 7:**
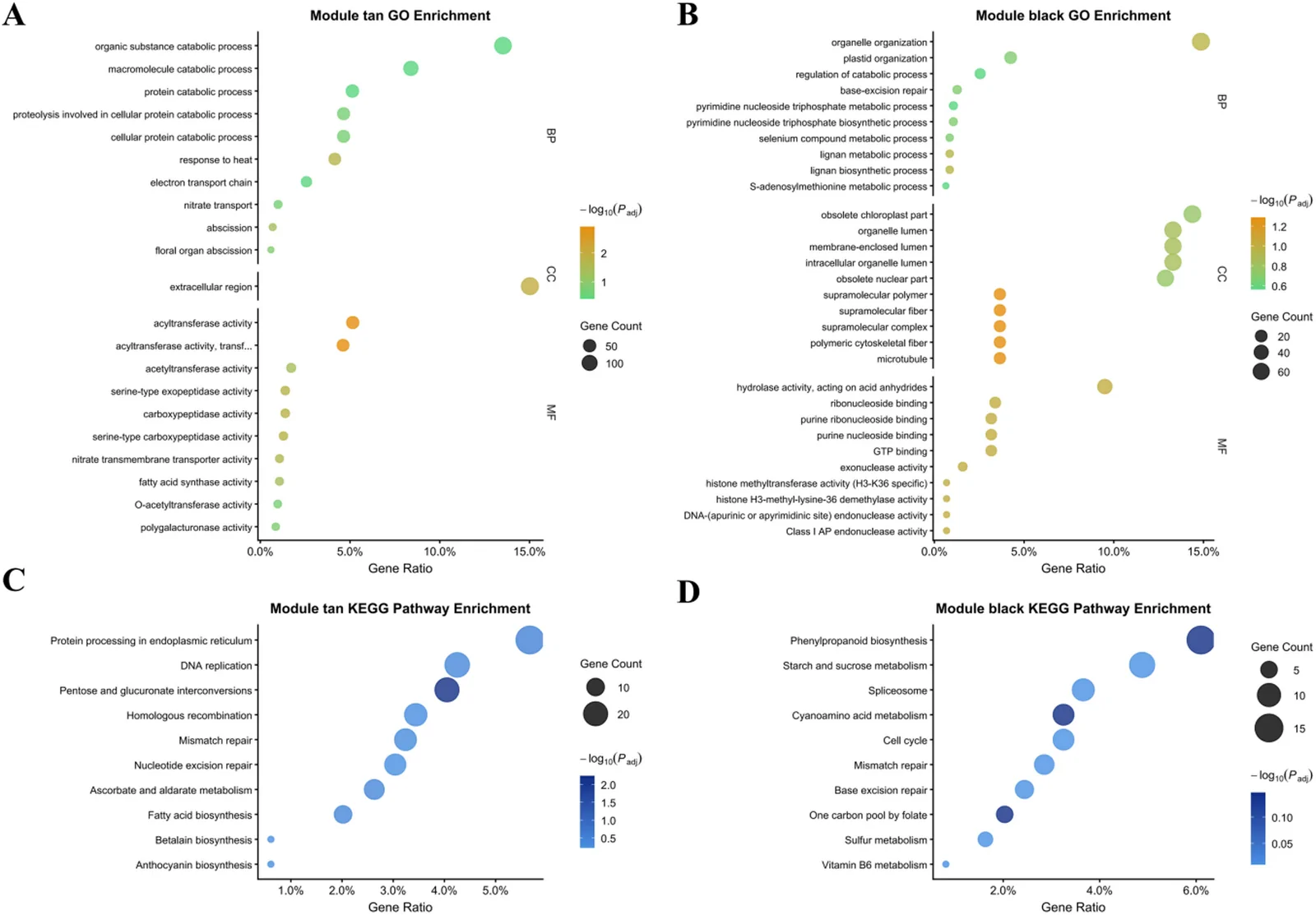
GO enrichment and KEGG enrichment analyses of Tan and Black module, respectively

The correlation of each characterized gene with sample treatment conditions was calculated, and two gene co-expression modules were identified (at |r|≥0.50, *p* < 0.05) (Fig. S6B). The Tan module (*r* = 0.89, *p* = 8e-08) was positively correlated with the germination rate, and the Black module (*r* = − 0.60, *p* = 0.004) was negatively correlated with the germination rate. To explore the functional classification and metabolic pathways of the responsive genes in different germination rates, we performed GO analysis of the genes in the Tan and Black modules. The genes in the Tan module were classified into 207 significantly enriched GO terms, including 74 Biological processes, 1 Cellular components, and 132 Molecular functions, which were mainly enriched in organic substance catabolic process (GO:1901575), macromolecule catabolic process (GO:0009057), extracellular region (GO:0005576), acyltransferase activity (GO:0016746), and hydrolase activity (GO:0016798) (Fig. 7A; Table S8). The genes in the Black module were classified into 1571 significantly enriched GO terms, including 1021 Biological processes, 312 Cellular components, and 431 Molecular functions, which were mainly enriched in organelle organization (GO:0006996), plastid organization (GO:0009657), microtubule (GO:0005874), hydrolase activity (GO:0016813), ribonucleoside binding (GO:0032549) (Fig. 7B; Table S9). The Tan module focuses on Pentose and glucuronate interconversions (ko00040), Protein processing in endoplasmic reticulum (ko04141), Fatty acid biosynthesis (ko00061), and Ascorbate and aldarate metabolism (ko00053) were enriched (Fig. 7C; Table S10). The Black module was mainly enriched in Cysteine and methionine metabolism (ko00270), Citrate cycle (TCA cycle) (ko00020), and Starch and sucrose metabolism (ko00500) (Fig. 7D; Table S11).

**Fig. 8:**
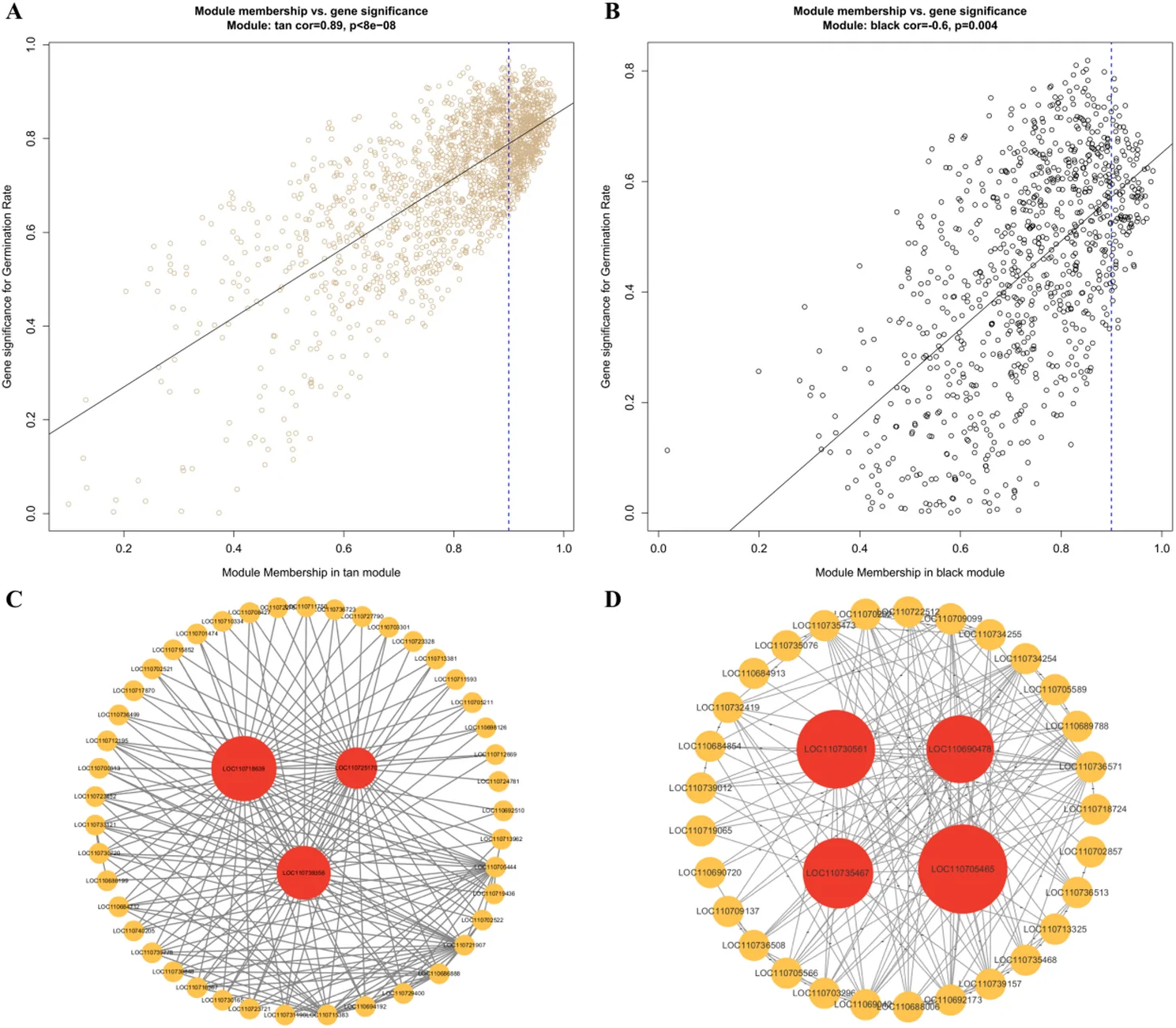
Scatter plots of gene significance (GS) versus module membership (MM) in Tan (**A**) and Black (**B**) modules. Candidate hub genes for Tan (**C**) and Black (**D**) obtained from interaction network analysis with known core genes

Scatter plots of GS and MM values were plotted for the Tan (Fig. 8A) and Black modules (Fig. 8B). Key genes were identified by applying thresholds of |GS| > 0.9 and |MM| > 0.9, resulting in the selection of 345 pivotal genes from the Tan module (Fig. 8C). Additionally, using more stringent criteria with |GS| > 0.7 and |MM| > 0.8, 42 key genes were screened from the Black module (Fig. 8D). The analysis network in Cytoscape 3.10.4 was subsequently used to select the top degree-ranked genes as hub genes. Finally the Tan module identified genes *LOC110718639*, *LOC110725170*, and *LOC110739356*. The Black module identified genes *LOC110730561*, *LOC110735467*, *LOC110690478*, and *LOC110705465*. Protein sequence comparison of hub genes from the identified modules was performed using the PlantTFDB database. The results revealed that the core genes in the Tan module belong to the C2H2, MYB, and bZIP transcription factor families. In contrast, the core genes in the Black module were classified into the FAR1, GeBP, and MYB transcription factor families. Functional annotation of these key candidate genes was further conducted through the TAIR database (Table S12).

### Gene expression validation by qRT-PCR analysis

To validate the RNA‑seq data, eight differentially expressed genes involved in seed storage, starch degradation, GA and ABA signaling, and transcriptional regulation were selected for qRT‑PCR analysis across the three genotypes at multiple time points after imbibition (Fig. S7). Overall, the expression patterns of these target genes measured by qRT‑PCR showed strong concordance with the transcriptome data, confirming the reliability and accuracy of the RNA‑sequencing results.

### Integrated regulatory model of quinoa seed germination

To systematically clarify the regulatory relationships among seed coat traits, hormone balance, transcriptional modules, and seed germination, we integrated Pearson correlation analysis, partial least squares path modeling (PLS-PM), and a schematic regulatory model to validate and visualize the core mechanisms.

Pearson correlation analysis was performed to assess the relationship between water absorption rate and GA_3_/ABA contents in each genotype during germination (Fig. S8). Water absorption rate was significantly positively correlated with GA_3_ content in *TF* (*r* = 0.92, *p* = 0.010) and *TS* mutants (*r* = 0.96, *p* = 0.003), but not in WT (*r* = 0.18, *p* = 0.737). No significant correlations between water absorption rate and ABA content were observed in any genotype. These results indicate GA_3_ may promote water uptake in *TF* and *TS* mutants, while ABA has no direct effect on water absorption.

PLS-PM analysis identified significant direct regulatory paths linking seed coat traits, hormone balance, and transcriptional modules to germination (Fig. S9; Table S13). Seed coat traits exerted a significant positive effect on MEpink (β = 0.66) and a significant negative effect on MEblack (β = − 0.57). The GA₃/ABA hormone ratio showed a strong positive influence on MEtan (β = 0.91) and a negative effect on MEblack (β = − 0.51). In turn, MEtan significantly promoted germination rate (β = 0.95), whereas MEpink, MEblack, and MEgrey showed no significant direct effects on germination. The model exhibited strong explanatory power, with R² = 0.861 for germination rate, indicating high reliability of the integrative framework. *R*² values for the four modules were 0.645 (MEpink), 0.917 (MEtan), 0.772 (MEblack), and 0.054 (MEgrey), respectively (Table S14). These results support a mechanistic regulatory model in which seed coat traits modulate germination primarily through the pink and black modules, while the GA₃/ABA ratio regulates germination mainly via the tan module. The high explanatory power confirms that the integrated model captures the major determinants of quinoa seed germination. A schematic model summarizing the regulatory framework of quinoa seed germination is shown in Fig. S10. This model illustrates how the integration of structural, hormonal, and transcriptional programs determines germination efficiency in quinoa.

## Discussion

### Association between seed coat structure and germination characteristics

The seed coat structure of quinoa, through the coordinated specialization of its multiple tissue layers, plays a crucial role in the physical and metabolic regulation of seed dormancy and germination [48]. As the primary interface between the seed and the external environment, the structure and composition of the seed coat directly influence water penetration and gas exchange [49]. The results of this study demonstrate that wild-type seeds germinated earliest and at the fastest rate, while also exhibiting the highest water absorption rate. This is consistent with their relatively thin palisade layer (13.31 μm) and intact cuticle structure (Fig. 2; Table 1). In contrast, the *TF* mutant displayed an overall thickening of the seed coat (36.20 μm), particularly a significant increase in the parenchyma tissue layer (11.78 μm). This structural alteration likely increased resistance to water penetration, leading to delayed germination and reduced water absorption (Fig. 1A, B). Although the total seed coat thickness of the *TS* mutant was comparable to that of the WT, its palisade layer was abnormally thickened (20.07 μm) and contained irregular intercellular spaces. Simultaneously, its parenchyma tissue layer was extremely thin (4.94 μm). This structural imbalance likely severely hindered uniform water absorption and embryo hydration, resulting in extremely delayed germination and a low final germination rate. These findings are consistent with previous studies in various plant species, in which the palisade layer has been regarded as a key structural layer related to mechanical constraint. Anatomically, thicker palisade layers are often associated with delayed radicle emergence, possibly by influencing water and gas permeability [41, 50, 51]. In quinoa, palisade layer thickness has been reported to correlate with seed dormancy intensity by affecting water uptake and ABA leaching [6, 52]. Although these structural observations support the notion that the palisade layer may function as a mechanical barrier, biochemical evidence such as lignin and suberin deposition remains to be further characterized in future investigations.

Beyond its role as a physical barrier, the parenchyma tissue layer also plays a critical metabolic regulatory function. As a living cellular tissue, this layer is essential for nutrient storage and transport to the developing embryo [53, 54]. The well-developed parenchyma tissues in WT and *TF* mutants likely facilitate the efficient transport of carbohydrates, amino acids, and hormone precursors, thereby meeting the metabolic demands of early germination [55, 56]. In contrast, the notably thinner parenchyma tissue in the *TS* mutant suggests limited nutrient transport capacity, which may constrain the energy supply to the growing radicle [57, 58]. Consequently, the *TS* mutant exhibits a restrictive seed coat structure characterized by both a strong physical barrier and insufficient metabolic support. The role of parenchyma tissue in symplastic nutrient transport has been demonstrated in other species [59, 60], and its reduction in the *TS* mutant provides a structural explanation for the observed germination delay. Furthermore, the combination of a thickened palisade layer and a thin parenchyma layer in the *TS* mutant may not only impede the diffusion of water and oxygen but also disrupt the exchange of germination inhibitors and promotive signals [2, 61]. These ultrastructural features highlight the crucial role of seed coat architecture and its physical constraints in regulating the timing of germination.

### The core regulatory role of endogenous hormone homeostasis in seed germination

Hormones function as pivotal molecular switches in seed germination, with the antagonistic balance between GA_3_ and ABA serving as the core mechanism regulating germination initiation and progression [48, 62–64]. The results of this study indicate significant differences among the three genotypes in the dynamics of GA₃ and ABA contents as well as their ratios (Fig. 3), which closely align with their germination phenotypes. In wild-type seeds, GA₃ rapidly accumulated and peaked during the early imbibition stage (8 h), concurrently with the highest GA₃/ABA ratio. This rapid hormonal reprogramming is directly associated with their efficient germination initiation. In contrast, the *TF* mutant exhibited delayed and dysregulated hormone dynamics: the GA₃ accumulation peak occurred later and was lower in magnitude, while the ABA peak was higher and degraded more slowly, resulting in a delayed and sustained lower GA₃/ABA ratio. This provides a physiological explanation for its delayed germination. The *TS* mutant displayed the most severe hormonal imbalance, characterized by persistently low GA₃ levels throughout imbibition, coupled with the highest basal and peak ABA levels. Consequently, the GA₃/ABA ratio remained extremely low. This typical “high ABA, low GA” hormonal profile is closely associated with phenotypes of deep dormancy or strong germination inhibition [65].

It is noteworthy that while changes in absolute hormone levels are important, recent research increasingly emphasizes the critical role of hormone signal perception and transduction efficiency [66]. For instance, the abundance and activity of core components in the ABA signaling pathway, such as PYL/PYR/RCAR receptors, PP2C phosphatases, and SnRK2 kinases, as well as the degradation rate of DELLA proteins in the GA signaling pathway, may directly influence seed sensitivity to hormones [54]. The exogenous hormone treatment experiments in this study provide supporti*ve phenotypic* evidence. Exogenous GA₃ significantly alleviated the germination defects of the *TS* mutant, while different genotypes showed distinct sensitivities to exogenous ABA inhibition, with sensitivity negatively correlated with their endogenous ABA levels (Fig. S1A, B). These results imply that the impaired germination in the *TS* mutant may be related to insufficient endogenous GA_3_ biosynthesis and potentially reduced efficiency in GA signaling. Meanwhile, the relatively high endogenous ABA level in the *TS* mutant may cause partial saturation or feedback regulation in the ABA response, thus weakening its sensitivity to exogenous ABA. Additionally, crosstalk among GA, ABA, and other hormones such as IAA, BRs, and ETH has been recognized as an important regulatory module in fine-tuning seed germination [25, 67]. Since gene expression changes underlying these hormone-induced phenotypic responses were not examined in the present study, future investigations combining hormone quantification with expression analysis of core GA/ABA signaling components will help to further clarify the detailed hormonal regulatory mechanisms underlying germination differences in quinoa. 

### Transcriptomic insights into the multi-layered gene expression regulatory network

Transcriptomic analysis provides molecular-level explanations for the observed phenotypic differences [68]. Principal component analysis revealed that the germination process is accompanied by extensive transcriptional reprogramming, with genotypic differences becoming more pronounced over time (Fig. 4A). Differential expression gene analysis indicated that the *TS* mutant exhibited far fewer transcriptional changes between 0 and 12 h compared to the WT and *TF* mutants, whereas a substantial number of DEGs emerged between 12 and 24 h (Fig. 4B). This suggests that key transcriptional activation events associated with germination are severely delayed in the *TS* mutant, aligning with its sluggish physiological initiation process.

GO and KEGG enrichment analyses revealed distinct, germination‑related metabolic and signaling programs across genotypes (Fig. 5). In WT, DEGs were strongly enriched in plant hormone signal transduction, MAPK signaling pathway, and cell wall metabolism, consistent with efficient activation of cell elongation, division, and germination‑promoting stress responses that support rapid and normal germination [69, 70]. By contrast, the moderately germination‑defective *TF* mutant exhibited prominent enrichment in carbohydrate and lipid metabolism, which likely represents a compensatory metabolic strategy to mobilize energy reserves and synthesize structural components required for delayed but progressive germination [71]. The severely impaired *TS* mutant displayed a unique profile marked by enrichment in photosynthesis- and ribosome‐related pathways. Enhanced photosynthesis‐related gene expression suggests disrupted energy homeostasis, redox balance, and carbon metabolism, which collectively impair germination vigor [72]. Meanwhile, enrichment of ribosomal pathways indicates either globally repressed metabolic activity associated with deep germination arrest or an active inhibitory mechanism that restricts translation. Together, these expression patterns reveal broad dysregulation of energy, hormone, and translational programs, which likely contribute to the severe germination defects in *TS* [73]. Future studies integrating proteomic and functional validation will be required to establish causal relationships.

Weighted gene co-expression network analysis further identified gene modules that were significantly positively correlated (Tan module) and negatively correlated (Black module) with germination rate (Figs. 6 and 7). Genes within the Tan module were enriched in functions related to macromolecule catabolism and the extracellular region, which are highly relevant to key events during seed germination such as the hydrolysis of stored reserves and cell wall loosening to facilitate radicle emergence [74, 75]. In contrast, the Black module was associated with organelle organization and microtubule function, and its upregulation may correspond to a state of germination inhibition or dormancy maintenance [76]. Hub genes from key modules were indentified by combining WGCNA connectivity and biological relevance to seed germination (Table S12). These hubs include multiple transcription factor families, such as MYB, bZIP, and FAR1, all of which have been widely reported to regulate seed germination via GA/ABA signaling and stress responses [77]. For the MYB family, rice R3-MYB transcription factors *OsTCL1* and *OsTCL2* modulate ABA synthesis, metabolism, and signal transduction, thus controlling endogenous ABA levels and seed germination process under abiotic stress [78]. MYB genes identified here may function similarly in regulating quinoa germination through ABA-dependent pathways. bZIP family members, such as ABI5 and ABFs, act as core repressors of germination in ABA signaling [79, 80]. As phyA pathway components, FAR1/FHY3 integrate light and ABA signals by activating ABI5 to inhibit germination [81]. The identification of these hub genes provides important targets for further investigation into the key regulatory nodes controlling germination in quinoa. 

### Limitations and future perspectives

This study reports the first integrated analysis of quinoa seed germination, combining seed coat electron microscopy, hormone profiling, transcriptomics, and WGCNA to unravel the coordinated roles of seed coat structure, hormone regulation, and transcriptional networks, while nominating key hub genes for functional validation. However, several limitations should be acknowledged. First, transcriptomic profiling was limited to three time points, restricting resolution of transcriptional dynamics during critical early (2–8 h) and later (24–48 h) germination phases. Future work should adopt denser temporal sampling to capture the full spectrum of transcriptional reprogramming. Second, despite stable germination phenotypes, the causal EMS-induced mutations in the TF and TS mutants remain uncharacterized, precluding distinction between primary genetic effects and secondary regulatory responses. Positional cloning or whole-genome resequencing will be critical to establish definitive genotype–phenotype links. Third, while GA_3_ and ABA dynamics were quantified, the contributions of other germination-relevant hormones (e.g., ethylene, cytokinins) were not assessed; multi-hormone profiling is needed to elucidate complex hormonal crosstalk. Fourth, ultrastructural differences in the seed coat were observed, but their association with biochemical components such as lignin and cutin remains untested. Systematic biochemical and structural characterization of the seed coat will facilitate quantitative models linking these traits to germination efficiency. Finally, the functional significance of candidate hub genes required experimental validation through genetic approaches such as gene editing, overexpression, and complementation assays in quinoa. Integrating these efforts with proteomic and metabolomic data will further enable the construction of a multi‑layered regulatory network from genes to phenotypes.

## Conclusion

In summary, this study systematically demonstrates that germination variation among quinoa genotypes results from the integration of seed coat architecture, endogenous hormone dynamics, stress response capacity, and genome-wide transcriptional regulation. The wild-type displayed coordinated structural and physiological characteristics that support efficient germination. The *TF* mutant exhibited adaptive structural and metabolic modifications associated with delayed germination. In contrast, the *TS* mutant showed impaired germination, which can be attributed to altered seed coat morphology, disrupted hormone homeostasis, and delayed transcriptional activation. These findings contribute to a more comprehensive understanding of the regulatory mechanisms underlying quinoa seed germination and provide a basis for further molecular studies aimed at improving germination-related traits.

## Supplementary Information

Supplementary Material 1.

Supplementary Material 2.

Supplementary Material 3.

Supplementary Material 4.

Supplementary Material 5.

Supplementary Material 6.

Supplementary Material 7.

Supplementary Material 8.

Supplementary Material 9.

Supplementary Material 10.

Supplementary Material 11.

Supplementary Material 12.

Supplementary Material 13.

## Data Availability

The raw sequencing data supporting the findings of this study have been deposited in the NCBI Sequence Read Archive (SRA) under the BioProject accession number PRJNA1344799 (https://www.ncbi.nlm.nih.gov/bioproject/PRJNA1344799).

## Data availability

The plant materials used in this study were provided by Shanxi Jiaqi Agricultural Technology Co., Ltd. Taiyuan, China.
