# Supplementary material for: Deciphering germination rate differences in quinoa genotypes via integrated phenotypic, structural, and transcriptomic approaches
Source: BMC Plant Biol. 2026 Apr 2;26:1556. doi: 10.1186/s12870-026-08676-7 (PMC13555991; doi:10.1186/s12870-026-08676-7)
Supplement: Supplementary file 1 [file 12870_2026_8676_MOESM13_ESM.docx]

**Supplementary Table 1. The primers used for qRT-PCR**

| **Primers** | **Gene_ID** | **Forward primer sequences** | **Reverse primer sequences** | **Amplicon size (bp)** |
| --- | --- | --- | --- | --- |
| *Gene1* | *LOC110728527* | GATCGATTTGGTTTATCGAAC | GTTGCGCAGCGGCGACC | 142 |
| *Gene2* | *LOC110685584* | GTCGACGTCGATGATGGTTC | GAAGAACAGGAAGAAAAAGC | 125 |
| *Gene3* | *LOC110739855* | GATTCATGCAATAAGCAAGG | CGTTGGAAGGAGAAGTGGT | 135 |
| *Gene4* | *LOC110730263* | GCTTCGAAGGTGCCGGTG | GTTGGTCTCCCTGGAATGG | 126 |
| *Gene5* | *LOC110733591* | GCTCACGTAGTGACTGAGAGT | CGTTTGCCACCGTGGGTG | 128 |
| *Gene6* | *LOC110729743* | GATGACACACAACTTGTATTTGA | GTTGGTCTCCCTGGAATGG | 138 |
| *Gene7* | *LOC110688388* | GATAGAAGACCACTACCCCCT | GTTGGTCTCCCTGGAATGG | 139 |
| *Gene8* | *LOC110684025* | GATAGAAGACCTCTACCCCCT | GTTGGTCTCCCTGGAATGG | 142 |
| *CqACT* | *LOC110715281* | AGCTTCTTGACGAAATGGGTG | ACAACTCCTCACCTTCTCATGG |  |

**Supplementary Table 2. Summary of RNA-seq data quality metrics across all experimental samples.**

| **Sample name** | **Number of filtered reads** | **Uniquely mapped** | **Total number of bases** | **Total number of filtered bases** | **Q20(%)** | **Q30(%)** |
| --- | --- | --- | --- | --- | --- | --- |
| A0hTF_1 | 43501556 | 36556483 (95.10%) | 6970927684 | 6568734956 | 97.54 | 93.04 |
| A0hTF_2 | 42822252 | 33739551 (95.27%) | 6873681268 | 6466160052 | 97.65 | 93.31 |
| A0hTF_3 | 40730090 | 36866263 (95.13%) | 6542056276 | 6150243590 | 97.2 | 92.56 |
| A0hTS_1 | 40288710 | 33545991 (96.04%) | 6487781742 | 6083595210 | 97.46 | 92.95 |
| A0hTS_2 | 43158918 | 38535089 (95.90%) | 6938084278 | 6516996618 | 97.59 | 93.28 |
| A0hTS_3 | 42547828 | 32280671 (96.07%) | 6877526332 | 6424722028 | 97.51 | 93.11 |
| A0hW_1 | 41068398 | 38157058 (94.71%) | 6624574152 | 6201328098 | 97.58 | 93.23 |
| A0hW_2 | 37999228 | 37886700 (94.83%) | 6090591644 | 5737883428 | 97.12 | 92.54 |
| A0hW_3 | 41441382 | 35648925 (94.94%) | 6639023946 | 6257648682 | 97.28 | 92.75 |
| A12hTF_Y1 | 41941166 | 38430717 (96.06%) | 6727378878 | 6333116066 | 97.51 | 93.27 |
| A12hTF_Y2 | 40166204 | 37208741 (96.00%) | 6442953164 | 6065096804 | 97.44 | 92.72 |
| A12hTF_Y3 | 44695206 | 41592971 (96.01%) | 7168527794 | 6748976106 | 97.72 | 93.36 |
| A12hTS_Y1 | 41721378 | 34775021 (94.50%) | 6705946240 | 6299928078 | 97.5 | 93.02 |
| A12hTS_Y2 | 33758558 | 37055725 (94.41%) | 5436850734 | 5097542258 | 97.13 | 91.97 |
| A12hTS_Y3 | 45234800 | 36628333 (94.39%) | 7273518396 | 6830454800 | 97.27 | 92.29 |
| A12hW_Y1 | 36675532 | 38602629 (96.21%) | 5890551072 | 5538005332 | 97.44 | 93.21 |
| A12hW_Y2 | 42060648 | 31282719 (96.16%) | 6747869880 | 6351157848 | 97.64 | 93.53 |
| A12hW_Y3 | 35278806 | 41656253 (96.14%) | 5665962732 | 5327099706 | 97.37 | 92.97 |
| A24hTS_Y1 | 43915488 | 40560402 (96.03%) | 7065012462 | 6631238688 | 97.66 | 93.26 |
| A24hTS_Y2 | 42585558 | 39539698 (96.08%) | 6850227646 | 6430419258 | 97.7 | 93.24 |
| A24hTS_Y3 | 45062668 | 41685854 (95.92%) | 7242056036 | 6804462868 | 97.59 | 93.07 |

**Fig. S1.** Germination percentage of WT, *TF*, and *TS* mutants under treatment with (A) 100 μM ABA, (B) 200 μM GA₃, (C) 150 mM NaCl, and (D) 10% PEG-6000. The experiment was performed with three independent replicates.

**Fig. S2.** Venn diagram analysis showing shared and unique DEGs. (A) DEGs among genotypes at 0 h; (B) DEGs in 0 h vs 12 h and 0 h vs 24 h across WT, *TF*, and *TS* mutant.

**Fig. S3.** (A) GO and (B) KEGG pathway enrichment analyses of DEGs between WT and *TF* at 12 h of germination. (C) GO and (D) KEGG pathway enrichment analyses of DEGs between WT and *TS* at 12 h of germination. (E) GO and (F) KEGG pathway enrichment analysis of DEGs between *TS* and *TF* at 12 h of germination.

**Fig. S4.** Phylogenetic dendrogram of all 19 samples. The tree shows hierarchical clustering based on gene expression profiles. Samples with similar expression patterns are clustered closely together.

B

A

**Fig. S5.** Network topology analysis for soft-threshold powers. X-axis shows the weight parameter β. (A) scale-free topology fit index (R²). (B) mean gene connectivity.

**B**

**A**

**Fig. S6.** Module-trait correlations and associations. (A) Bootstrap 95% confidence intervals for module–germination rate correlations. Points denote observed correlation coefficients, horizontal lines represent 95% confidence intervals. Red points indicate modules with robust associations (confidence intervals excluding zero), black points indicate non-robust associations (confidence intervals including zero). (B) Module-germination rate relationships.

**Fig. S7.** Validation of the transcription levels for selected DEGs via RT-qPCR.

**Fig. S8.** Scatter plot matrix of water absorption rate, GA₃, and ABA contents. Pairwise relationships among water absorption rate, GA₃ and ABA are shown by genotype. Upper triangle panels show Pearson correlation coefficients. Lower triangle panels display LOESS smoothing curves. Diagonal panels represent density distributions. Data points are semi-transparent (alpha = 0.6).

**Fig. S9.** PLS-PM path diagram illustrating relationships among seed coat traits, hormone ratio, gene modules, and germination rate. Note: Black solid lines indicate significant paths (95% CI excluding zero), gray dashed lines indicate non-significant paths. Numbers on arrows are standardized path coefficients.

**Fig.S10.** Schematic model illustrating the regulatory mechanisms underlying germination differences among wild‐type (WT), *TF*, and *TS* quinoa genotypes.
